# The expression patterns and correlations of claudin-6, methy-CpG binding protein 2, DNA methyltransferase 1, histone deacetylase 1, acetyl-histone H3 and acetyl-histone H4 and their clinicopathological significance in breast invasive ductal carcinomas

**DOI:** 10.1186/1746-1596-7-33

**Published:** 2012-03-29

**Authors:** Xiaoming Xu, Huiying Jin, Yafang Liu, Li Liu, Qiong Wu, Yaxiong Guo, Lina Yu, Zhijing Liu, Ting Zhang, Xiaowei Zhang, Xueyan Dong, Chengshi Quan

**Affiliations:** 1 The Key Laboratory of Pathobiology, Ministry of Education, Bethune Medical College, Jilin University, Changchun, Jilin, China; 2 Department of Pathology, Jilin Oil Field General Hospital, Songyuan, Jilin, China; 3 Department of Pathology, The First Bethune Hospital of Jilin University, Changchun, Jilin, China; 4 Department of Pathology, The Third Bethune Hospital of Jilin University, Changchun, Jilin, China; 5 The Key Laboratory of Pathobiology, Ministry of Education, Bethune Medical College, Jilin University, 126 Xinmin street, Changchun, Jilin 130021, People's Republic of China

**Keywords:** Claudin-6, Histone deacetylase 1, Methyl-CpG binding protein 2, DNA methyltransferase 1, Breast invasive ductal carcinomas

## Abstract

**Background:**

Claudin-6 is a candidate tumor suppressor gene in breast cancer, and has been shown to be regulated by DNA methylation and histone modification in breast cancer lines. However, the expression of claudin-6 in breast invasive ductal carcinomas and correlation with clinical behavior or expression of other markers is unclear. We considered that the expression pattern of claudin-6 might be related to the expression of DNA methylation associated proteins (methyl-CpG binding protein 2 (MeCP2) and DNA methyltransferase 1 (DNMT1)) and histone modification associated proteins (histone deacetylase 1 (HDAC1), acetyl-histone H3 (H3Ac) and acetyl- histone H4 (H4Ac)).

**Methods:**

We have investigated the expression of claudin-6, MeCP2, HDAC1, H3Ac and H4Ac in 100 breast invasive ductal carcinoma tissues and 22 mammary gland fibroadenoma tissues using immunohistochemistry.

**Results:**

Claudin-6 protein expression was reduced in breast invasive ductal carcinomas (*P *< 0.001). In contrast, expression of MeCP2 (*P *< 0.001), DNMT1 (*P *= 0.001), HDAC1 (*P *< 0.001) and H3Ac (*P *= 0.004) expressions was increased. Claudin-6 expression was inversely correlated with lymph node metastasis (*P *= 0.021). Increased expression of HDAC1 was correlated with histological grade (*P *< 0.001), age (*P *= 0.004), clinical stage (*P *= 0.007) and lymph node metastasis (*P *= 0.001). H3Ac expression was associated with tumor size (*P *= 0.044) and clinical stage of cancers (*P *= 0.034). MeCP2, DNMT1 and H4Ac expression levels did not correlate with any of the tested clinicopathological parameters (*P *> 0.05). We identified a positive correlation between MeCP2 protein expression and H3Ac and H4Ac protein expression.

**Conclusions:**

Our results show that claudin-6 protein is significantly down-regulated in breast invasive ductal carcinomas and is an important correlate with lymphatic metastasis, but claudin-6 down-regulation was not correlated with upregulation of the methylation associated proteins (MeCP2, DNMT1) or histone modification associated proteins (HDAC1, H3Ac, H4Ac). Interestingly, the expression of MeCP2 was positively correlated with the expression of H3Ac and H3Ac protein expression was positively correlated with the expression of H4Ac in breast invasive ductal carcinoma

**Virtual slides:**

The virtual slide(s) for this article can be found here: http://www.diagnosticpathology.diagnomx.eu/vs/4549669866581452

## Background

Breast cancer is the most common cancer in women worldwide and the leading cause of death among women with cancer [1]. It has been estimated that 230, 480 women would be diagnosed with and 39, 520 women would die of cancer of the breast in 2011. http://seer.cancer.gov/statfacts/html/breast.html.

It is widely accepted that the loss of cell-to-cell adhesion in tumor-derived epithelium is necessary for the invasion of surrounding stromal elements and subsequent tumor metastasis [2]. The cell-to-cell adhesion of epithelial cells is primarily mediated through two types of junctions: adherens junctions and tight junctions. Tight junctions consist of three parts, depending on their distribution within the junction: transmembrane proteins (which include occludins, the claudin family and junctional adhesion molecules), cytoplasmic plaques (the zonula occludens family), and associated/regulatory proteins (Rho-subfamily proteins) [3].

The claudin family includes at least 27 members [4,5]. Claudin-6 contains four transmembrane domains, similar to other members of the claudin family [6]. Recent studies have demonstrated that epigenetic mechanisms are essential for claudin regulation [7-9]. The most studied epigenetic regulators are DNA methylation and histone modification [10]. MeCP2 is a methyl-CpG binding protein that represses gene transcription. DNA methyltransferases are crucial enzymes for hypermethylation of tumor suppressor genes [11]. DNA methyltransferase 1 (DNMT1) is the best known and studied member of the DNMT family [12]. Moreover, DNA methylation occurs in a complex chromatin network and is influenced by modifications in histone structure [13,14]. Histone deacetylase 1 (HDAC1), a zinc-dependent deacetylase, is a member of class I histone deacetylases, it is deregulated in many cancers and plays a crucial role in cell cycle progression and proliferation [15]. However, the expression levels of histone deacetylase 1 in breast invasive ductal carcinoma do not appear to have been studied.

We hypothesized that alterations in the expression levels of several epigenetic regulators, such as MeCP2, DNMT1, HDAC1, H3Ac and H4Ac, are responsible for the loss of claudin-6 in breast invasive ductal carcinomas. Here, we have analysed 100 breast invasive ductal carcinomas and 22 mammary gland fibroadenomas by immunohistochemistry. Our results demonstrate decreased claudin-6 expression in 75% of our cases of invasive breast carcinomas. Claudin-6 expression was found to be independent of the tumor subtype but was inversely correlated with lymph node metastasis.

## Methods

### Specimen collection

The breast specimens consisted of 100 invasive ductal carcinomas (IDC) and 22 fibroadenomas (FA) obtained during the period 2006 to 2010 from patients being treated at the Jilin Oil Field General Hospital in Songyuan, China. In 100 IDC cases, there were 15 tumor free mammary gland tissues which were employed as normal controls. The study was approved by the Ethics Committee of Jilin University. All specimens had been fixed in 4% buffered formalin and embedded in paraffin. The case diagnoses were based on the World Health Organization (WHO) classification of breast cancer [16]. The presence or absence of cancer metastasis was determined at the time of the operation. Material collection and the clinical features of the patients are described in Tables 1 and 2.

**Table 1:** Expression of CLDN6, MeCP2 and DNMT1 and clinicopathological characteristics in breast invasive ductal carcinoma patients

Variables	Number of cases (%)	Claudin-6			MeCP2			DNMT1		
		
		Positive (%)	Negative (%)	*P*	Positive (%)	Negative (%)	*P*	Positive (%)	Negative (%)	*P*
Breast invasive ductal carcinoma	100(100%)	25(25%)	75(75%)	< 0.001	88(88%)	12(12%)	< 0.001	69(69%)	31(31%)	0.001
Control	22(100%)	20 (91%)	2 (9%)		6 (27%)	16(73%)		7(32%)	15(68%)	
Age (year)										
≤ 40	20(20%)	5(25%)	15(75%)	1.000	16(80%)	4(20%)	0.251*	15(75%)	5(25%)	0.060
> 40	80(80%)	20(33%)	60(67%)		72(90%)	8(10%)		73(91%)	7(9%)	
Tumour size(cm)										
≤ 5	91(91%)	24(26%)	67(63%)	0.444*	80(88%)	11(12%)	1.000*	65(71%)	26(29%)	0.131
> 5	9 (9%)	1(11%)	8(89%)		8(89%)	1(11%)		4(44%)	5(56%)	
Differentiation										
well	25(25%)	9(36%)	16(64%)	0.142	21(84%)	4(16%)	0.488*	17(68%)	8(32%)	0.902
poor	75(75%)	16(21%)	59(79%)		67(89%)	8(11%)		50(67%)	25(33%)	
Clinical stage										
I	20(20%)	5(21%)	19(79%)	0.760	18(90%)	2(10%)	0.074*	10(50%)	10(50%)	0.113
II	46(46%)	10(22%)	36(78%)		37(80%)	9(20%)		35(76%)	11(24%)	
III-IV	34(34%)	10(29%)	24(71%)		33(97%)	1(3%)		23(68%)	11(32%)	
Lymph node metastasis										
Positive	48(48%)	7(15%)	41(85%)	0.021	43(90%)	5(10%)	0.640	34(71%)	14(29%)	0.972
Negative	52(52%)	18(35%)	34(65%)		45(87%)	7(13%)		37(71%)	15(29%)	

*MeCP2 *Methyl-CpG-binding proteins, *DNMT1 *DNA methyl transferase 1. * Fisher's exact text

**Table 2:** Expression of HDAC1, H3Ac and H4Ac and clinicopathological characteristics in breast invasive ductal carcinoma patients

Variables	Number of cases (%)	HDAC1			H3Ac			H4Ac		
		
		Positive (%)	Negative (%)	*P*	Positive (%)	Negative (%)	*P*	Positive (%)	Negative (%)	*P*
Breast invasive ductal carcinoma	100(100%)	67(67%)	33(33%)		90(90%)	10(10%)		79(79%)	21(21%)	0.243*
Control	22(100%)	3 (14%)	19(86%)	< 0.001	14(64%)	8 (36%)	0.004*	20(91%)	2 (9%)	
Age (year)										
≤ 40	20(20%)	8(40%)	12(60%)	0.004	18(90%)	2(10%)	1.000*	17(85%)	3(15%)	0.554*
> 40	80(80%)	59(74%)	21(26%)		72(90%)	8(10%)		62(78%)	18(22%)	
Tumour size(cm)										
≤ 5	91(91%)	62(68%)	29(22%)	0.472*	84(92%)	7(8%)	0.044*	74(81%)	17(19%)	0.089*
> 5	9 (9%)	5(56%)	4(44%)		6(67%)	3(23%)		5(56%)	4(44%)	
Differentiation										
well	25(25%)	8(32%)	17(68%)	< 0.001	24(96%)	1(4%)	0.444*	20(80%)	5(20%)	0.887
poor	75(75%)	59(79%)	16(21%)		66(88%)	9(12%)		59(79%)	16(21%)	
Clinical stage										
I	20(20%)	12(60%)	8(40%)	0.007	17(85%)	3(15%)	0.034*	18(90%)	2(10%)	0.227
II	46(46%)	38(83%)	8(17%)		45(98%)	1(2%)		37(80%)	9(20%)	
III-IV	34(34%)	17(50%)	17(50%)		28(82%)	6(18%)		24(71%)	10(29%)	
Lymph node metastasis										
Positive	48(48%)	40(83%)	8(17%)	0.001	43(90%)	5(10%)	1.000*	37(77%)	11(23%)	0.651
Negative	52(52%)	27(52%)	25(48%)		47(90%)	5(10%)		42(81%)	10(19%)	

*HDAC1 *histone deacetylase 1, *H3Ac *acetyl-Histone H3, *H4Ac*, acetyl-Histone H4. * Fisher's exact text

### Immunohistochemistry

Four-micrometer-thick tissue sections were cut from the paraffin-embedded blocks. Deparaffinization was performed using a solution containing xylene, and the sections were rehydrated with graded ethanol. The slides were placed in target retrieval solution (citrate buffer, pH 6.0) and boiled for 5 min in a microwave. After the samples were cooled for 30 min, endogenous peroxidase activity was inhibited by treatment with 3% H_2_O_2 _for 30 min. The sections were washed with PBS three times. After a 30-min protein block with normal goat serum or normal rabbit serum, the samples were incubated with the following antibodies overnight at 4°C: anti-claudin-6 (1:500; Santa Cruz Biotechnologies, Santa Cruz, CA, USA), anti-MeCP2 (1:400; 3456, Cell Signalling Technology, Inc., Boston, MA, USA), anti-DNMT1 (1:500; GTX116011, Gene Tex, Inc., USA), anti-HDAC1 (E47) (1:400; BS5576, Bioworld Technology, Inc., USA), anti-acetyl-histone H3 (K9) (1:400; BS8009, Bioworld Technology, Inc., USA), anti-acetyl-histone H4 (K12) (1:400; BS8010, Bioworld Technology, Inc., USA). Immunostaining was performed using the streptavidin-biotin-peroxidase complex. The biotin-conjugated secondary antibody was incubated for 30 min at room temperature. In addition, the colour reagent diaminobenzidine (Bios, Beijing, China) was used to visualize the bound antibody. The sections were counterstained with Mayer's haematoxylin.

### Evaluation of cellular phenotypes

The number of positive-staining cells showing brown staining on the cell membrane and/or cytoplasm (for claudin-6) and nucleus (for MeCP2, DNMT1, HDAC1, H3Ac and H4Ac) in 5 randomly-selected 400× microscopic fields was counted and the percentage of positive cells was calculated.

Claudin-6 expression in more than 10% of tumor cells was defined as high expression [17]. Immunostaining results for DNMT1 [18,19] and HDAC1 [20] were interpreted as high expression when > 20% of the tumor cells were stained. MeCP2 expression in more than 15% of tumor cells was defined as high expression. H3Ac and H4Ac expression in more than 40% of tumor cells was defined as high expression.

All immunohistochemical analyses were evaluated separately by two pathologists (C.S.Q and K.P.Q), and discordant results were reviewed to reach an agreement.

### Statistical analyses

Statistical analysis was performed using SPSS 15.0. The Student's T tests and the Mann-Whitney *U *test were performed. Comparisons between sample groups were analysed for statistical significance using the *Χ*^2^-test and Fisher's exact test. The Spearman's correlation test was used to examine the correlation among claudin-6, MeCP2, DNMT1, HDAC1, H3Ac and H4Ac levels. All *P *values quoted are two-sided and *P *< 0.05 was considered statistically significant.

## Results

### Population and tumor characteristics

The clinicopathological characteristics of the patients are summarised in Tables 1 and 2. The mean age of patients with breast invasive ductal carcinoma was 50.5 years (range: 31-87 years). Negative nodes were found in 52 cases. A total of 31 cases had between 1 and 3 metastatic nodes, and 17 cases had more than 3 positive nodes.

### Protein expression in fibroadenomas and normal breast tissue

**Figure 1:**
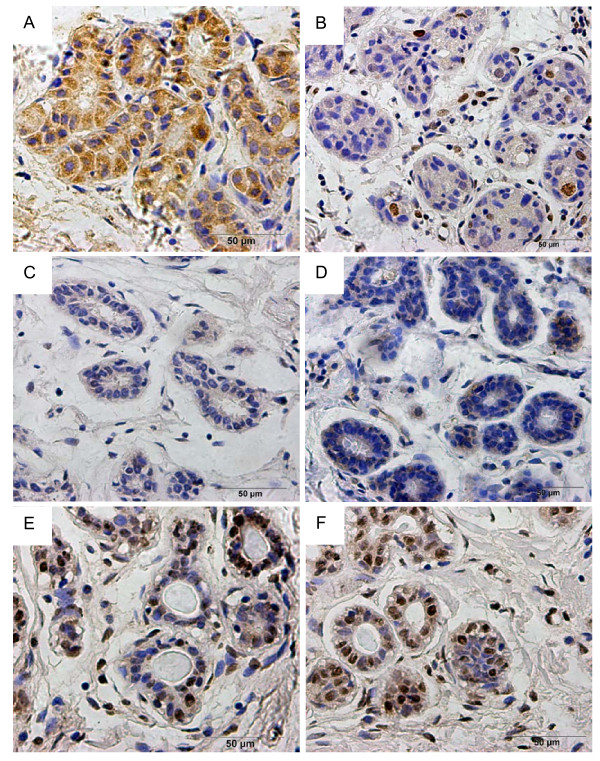
**Protein expression in normal breast tissue by immunohistochemistry**. (A), claudin-6 was expressed; (B), MeCP2; (C), DNMT1; (D), HDAC1 were expressed at low levels; (E), H3Ac was highly expressed; (F), the strong expression of H4Ac (400×).

**Figure 2:**
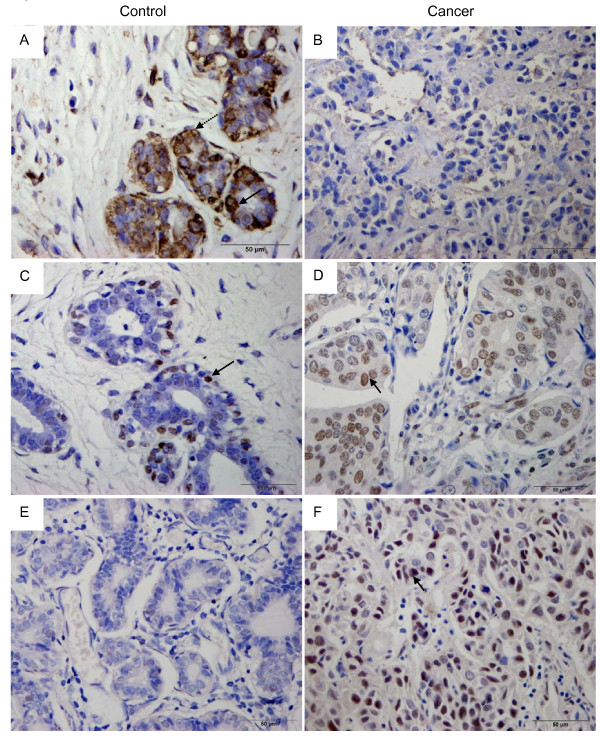
**Representative photomicrographs**. Immunohistochemical expressions for claudin-6, methy-CpG binding protein 2 (MeCP2) and DNA methyltransferase 1 (DNMT1) in breast invasive ductal carcinomas (IDC) and fibroadenomas (FA) (×400). (A) claudin-6 was expressed in breast adenoma cells. Note: the staining on membrane (dotted arrow); the staining in cytoplasm (solid arrow). (B) claudin-6 was weakly expressed in IDC cells. (C) MeCP2 immunoreactivity was moderate in the FA tissues (arrow). (D), (F) MeCP2 and DNMT1 were strongly expressed in breast IDC cells, especially at the nuclear (arrow). (E) DNMT1 immunoreactivity was not detected in the breast FA tissues.

**Figure 3:**
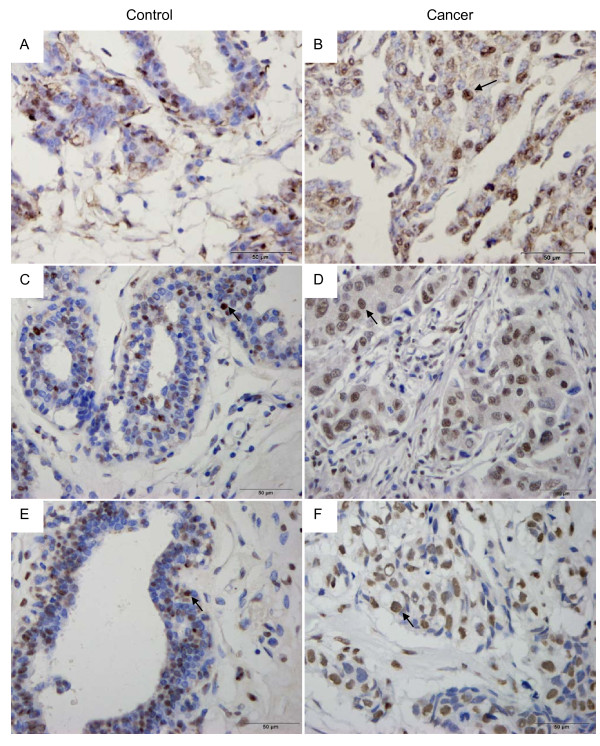
**Representative photomicrographs**. Immunohistochemical expressions for histone deacetylase 1 (HDAC1), acetyl-histone H3 (H3Ac) and acetyl-histone H4 (H4Ac) in breast invasive ductal carcinomas (IDC) and fibroadenomas (FA) (×400). (A), (C), (E) breast fibroadenomas; (B), (D), (F) breast invasive ductal carcinoma. Immunostained for HDAC1 (A, B), H3Ac (C, D) or H4Ac (E, F). nuclear staining (arrow).

We examined the expression of claudin-6, DNMT1, MeCP2, HDAC1, H3Ac and H4Ac in normal breast tissue adjacent to the carcinomas and in FA tumors (Figures 1, 2 and 3). There was no difference in the expression of these proteins between normal tissues adjacent to the tumors and in breast fibroadenomas (Table 3). Consequently, we chose the fibroadenomas as a control tissue.

**Table 3:** Expression of CLDN6, MeCP2, DNMT1, HDAC1, H3Ac and H4Ac in breast fibroadenoma tissues and normal adjacent tissues

Variables	Fibroadenoma tissues	Normal adjacent tissues	*P*
Expression Claudin-6 (%) Median (P25, P75)	28 (21, 47)	30 (25, 47)	0.953**^a^**
Expression MeCP2 (%) Median (P25, P75)	15 (10, 42)	14 (13, 43)	0.813**^a^**
Expression DNMT1 (%) Median (P25, P75)	6 (3, 14)	6 (4, 16)	0.442**^a^**
Expression HDAC1 (%) Median (P25, P75)	10 (7, 21)	10 (5, 21)	0.575**^a^**
Expression H3Ac (%) Mean ± SD	44.2 ± 19.8	45.6 ± 22.4	0.843**^b^**
Expression H4Ac (%) Mean ± SD	63.3 ± 18.2	61.8 ± 14.7	0.791**^b^**

^a ^Mann-Whitney *U *test; ^b ^t-student's test

### The expression of claudin-6 was reduced in breast invasive ductal carcinomas and was correlated with lymph node metastasis

In this study, claudin-6 was evaluated in the cytoplasm or membranes in 100 cases of breast IDCs and 22 breast FA samples. Positive expression of claudin-6 protein was found in 25% (25/100) of breast IDCs and in 91% (20/22) of breast FA cases (Table 1). The expression rate of claudin-6 in IDCs [median = 6.0% (P_25 _= 4.0%, P_75 _= 11.8%)] was lower than the rate in FAs [28.6% (20.0%, 47.2%)] (Mann-Whitney *U *test, *P *< 0.001) (Figure 2A, B). As shown in Table 1 the expression of claudin-6 was not correlated with age (*P *= 1.000), tumor size (*P *= 0.444), clinical stage (*P *= 0.760) or differentiation (*P *= 0.142) and was inversely correlated with lymph node metastasis of the breast invasive ductal carcinomas (*P *= 0.021).

### The expression of MeCP2 and DNMT1 was increased in breast invasive ductal carcinomas

The nuclear staining of MeCP2 and DNMT1 was strong in IDC cells and weak in FA adenocytes. MeCP2 was expressed in 88% (88/100) of breast IDC samples. The expression rate was [67.0% (43.5%, 76.0%)] in IDC samples. Cells were positive for MeCP2 in 27% (6/22) of breast FA cases. The expression rate was [15.5% (11.5%, 42.3%)]. We conclude that MeCP2 expression is significantly high (Figure 2C, D) in breast IDC samples (Mann-Whitney *U *test, *P *< 0.001). The expression level of DNMT1 was high in most breast IDCs but was low in breast FAs (Figure 2E, F). The positive expression of DNMT1 in the 100 breast cancer samples was 69% (69/100), which was significantly greater than that in the breast FA tissues 32% (7/22), (*P *= 0.001). The average expression rate of DNMT1 in breast IDCs (25.5% ± 10.3%) was higher than the rate in breast FAs (7.1% ± 5.1%; t = 6.299; *P *< 0.001). As shown in Table 1, the expression of MeCP2, DNMT1 did not show any relationship to the clinicopathological characteristics of breast IDCs.

MeCP2 expression did not correlate with age (*P *= 0.251), tumor size (*P *= 1.000), clinical stage (*P *= 0.074), differentiation (*P *= 0.488) or lymph node metastasis (*P *= 0.640). The expression of DNMT1 was not correlated with age (*P *= 0.060), tumor size (*P *= 0.131), clinical stage (*P *= 0.113), differentiation (*P *= 0.902) or lymph node metastasis (*P *= 0.972).

### The expression of HDAC1 was positively correlated with the poor differentiation and lymph node metastasis of breast invasive ductal carcinomas

In the present study, the expression of HDAC1 was found in 67% (67/100) of breast IDCs, the expression rate was [26.0% (13.3%, 39.0%)]. The expression of HDAC1 was found in 14% (3/22) of breast FAs, the expression rate was [11.0% (7.8%, 21.5%)] (Figure 3A, B). The average expression rate of HDAC1 in breast IDCs was significantly higher than that in breast FAs (Mann-Whitney *U *test, *P *= 0.002). As shown in Table 2 the expression of HDAC1 was positively correlated with the poor differentiation (*P *< 0.001), older age (*P *= 0.004) clinical stage (*P *= 0.007) and lymph node metastasis (*P *= 0.001), but was not related to the tumor size (*P *= 0.472) in the breast cancer tissues.

### Increased expression of H3Ac was correlated with tumor size and clinical stage in breast invasive ductal carcinomas

The expression of H3AC was found in 90% (90/100) of breast IDCs, whereas the expression was found in 64% (14/22) of breast FAs. The difference was significant (*P *= 0.004) (Figure 3C, D). The average expression rate of H3Ac in breast IDCs was (63.1% ± 20.0%), which was significantly higher than that in breast FAs (44.6% ± 20.2%; t = 3.927, *P *< 0.001). The expression of H3AC was not correlated with age (*P *= 1.000), differentiation (*P *= 0.444) or lymph node metastasis (*P *= 1.000). The expression of H3AC was higher in small tumors (size ≤ 5 cm) than in large tumors (size > 5 cm) (*P *= 0.044) and was lower in clinical stages I and III-IV than that in clinical stage II (*P *= 0.034). The expression of H4AC was slightly elevated in FA samples compared to IDC samples. However, this difference was not statistically significant (*P *= 0.24, Fisher's exact text) (Figure 3E, F). H4AC expression was not correlated with any clinicopathological parameter (Table 2).

### MeCP2, H3Ac and H4Ac may be concurrently expressed in breast invasive ductal carcinomas

We investigated the correlation among claudin-6, MeCP2, DNMT1, HDAC1, H3AC and H4AC in 100 breast invasive ductal carcinomas using Spearman's correlation test. Although we did not find a correlation between claudin-6 and MeCP2, DNMT1, HDAC1, H3AC and H4AC, we found that the expression of MeCP2 was positively correlated with the expression of H3Ac (correlation coefficient = 0.206; *P *= 0.040). Moreover, the expression of H3Ac was significantly positively correlated with the nuclear expression of H4Ac (correlation coefficient = 0.292; *P *= 0.001). The detailed results of the analysis are described in Table 4.

**Table 4:** Correlation between the expression of claudin-6, MeCP2, DNMT1, HDAC1, H3AC and H4AC

		Expression of MeCP2	Expression of DNMT1	Expression of HDAC1	Expression of H3Ac	Expression of H4Ac
Expression of claudin-6	Correlation coefficient	-0.033	-0.147	-0.091	-0.011	-0.072
	P value	0.746	0.145	0.369	0.917	0.478
Expression of MeCP2	Correlation coefficient		0.060	0.019	0.206	0.162
	P value		0.552	0.855	**0.040**	0.107
Expression of HDAC1	Correlation coefficient			0.023	0.126	-0.025
	P value			0.821	0.210	0.804
Expression of DNMT1	Correlation coefficient				0.072	0.110
	P value				0.473	0.278
Expression of H3Ac	Correlation coefficient					0.292
	P value					**0.001**

We analysed the association between the loss of claudin-6 and the protein expression of the potential transcriptional repressors MeCP2, HDAC1, H3AC and H4AC. These results suggest that histone modifications might account for claudin-6 inhibition in breast IDC. No correlations were detected between the loss of claudin-6 and the upregulation of MeCP2 (*P *= 0.746) and H3AC (*P *= 0.917) in IDC samples.

## Discussion

In a previous study, we showed that the protein and gene expression of claudin-6 was low or undetectable in human and rat mammary cancer cell lines [21], and the cell growth, migration and invasion were inhibited by overexpression of claudin-6 in breast cancer MCF-7 cells. These results suggested that claudin-6 is a tumor repressor that inhibits malignant progression of breast cancer [22]. Claudin-6 expression is inactivated by aberrant CpG island DNA hypermethylation in its promoter region in the breast cancer cell MCF-7 [23], suggesting that DNA methylation may have an important role in regulation of claudin-6 expression in breast cancer. The most studied epigenetic regulators are DNA methylation and histone modification [10]. DNA methylation can cause gene silencing by interfering with the interactions between transcriptional activators and target-binding sites on genomic DNA, condensing the chromatin to alter DNA accessibility, and recruiting methyl-CpG binding proteins, which mediate downstream biological effects [24]. However, whether or not methylation associated proteins and histone modification proteins act to repress claudin-6 expression and how they influence the clinicopathological characteristics of IDC tumors is not yet understood. Consequently, our main goal in this study was to analyze the importance of MeCP2, DNMT1, HDAC1, H3Ac and H4Ac in the down-regulation of claudin-6 in breast IDC.

We wanted to find the normal mammary gland tissue as a normal control, but it is rarely available for research in China. In this study, 15 (15%) of breast cancer cases had tumor free mammary gland tissue adjacent to the tumor samples. Since we regarded this as too small an amount to be used as a control, we then chose tissue from fibroadenomas for this purpose. Hua et al. studied that the expression of fibroblast activation protein-alpha (FAP-α) and Calponin was a novel marker for pathologically diagnosing whether DCIS had microinvasion, and FAP-α promoted the formation of microemboli, which facilitated the metastasis of breast cancer [25]. We also compared the expression of proteins we studied in tissue adjacent to tumors and in fibroadenomas. There was no difference in the expression of proteins between normal tissues adjacent to the 15 tumors, and breast fibroadenomas (*P *> 0.05; Table 3). Consequently chose the fibroadenomas as a control tissue.

In this study, we have shown that the expression of claudin-6 was observed in both membrane and cytoplasm/membrane in the FA and IDC of the breast, similar to previous studies on the claudin-6 in atypical teratoid/rhabdoid tumors [26], claudin-1, and claudin-7 in colonic and renal carcinomas, respectively [27,28]. This observation suggests that in breast IDCs and FAs, claudin-6 has an abnormal cellular localization, and was not restricted to cell-cell boundaries. Subsequent evaluation by immuno- histochemistry of 25 (25%) IDC samples and 20 (91%) FA samples showing positive immunoreactivity for claudin-6, showed that the expression of claudin-6 was significantly reduced in breast IDC tissues (*P *< 0.001). This result indicates that the reduced expression of claudin-6 may be an important molecular event in the development of breast cancers. Next, we investigated the correlation between claudin-6 expression and the clinicopathological characteristics of breast IDCs. We found that the expression of claudin-6 was negatively correlated with lymphatic metastasis of breast IDCs. This observation suggested low claudin-6 expression might facilitate lymph node invasion by tumor cells, and distant metastasis. On the one hand, the down-regulation of claudin-6 might result in abnormal proliferation, poor differentiation and decrease apoptosis, and played a role in mammary epithelial cell malignant transformation in the breast cancers; On the other hand, the down-regulation of claudin-6 may also lead to dysfunction of tight junctions, resulting in loss of cell-cell adhesion and polarity, causing tumor cells invasion and metastasis.

We have previously investigated the possibility that the expression of claudin-6 was negatively correlated with the hypermethylation promoter of claudin-6 in breast cancer tissues (data unpublished), suggesting that the down-regulation of claudin-6 is associated with its DNA methylation. DNA methylation is mediated by DNA methyltransferases (DNMTs) that catalyze the transfer of the methyl group from S-adenosyl L-methionine (SAM) to the cytosine in CpG dinucleotide [29]. Maintenance of methylation patterns is mediated by DNMT1. Altered levels of DNMT1 expression have frequently been associated with several types of tumors. DNMT1 expression was upregulated in pancreatic cancer cells, possibly associated with the progression of disease symptoms in pancreatic carcinoma [18]. In the present study, DNMT1 was more highly expressed in breast IDCs than nonmalignant tissues, but positive expression of DNMT1 was not associated with any clinical parameters. The Spearman's correlation test showed that claudin-6 expression was not correlated with the DNMT1 expression, but there were 56 cases of IDC in which the expression of claudin-6 and DNMT1 showed a reverse trend. The results show that the increased expression of DNMT1 plays an important role in the progression of breast IDC and that claudin-6 is partly inhibited by DNMT1.

MeCP2, the most studied member of the methyl-CpG binding domain proteins (MBDs), binds methylated DNA, and also serves as a transcriptional repressor [30]. Therefore, we considered whether claudin-6 expression might be related to the expression of MeCP2 in breast IDCs. Our results show that MeCP2 protein expression was statistically significantly higher in breast IDC specimens than in non-neoplastic lesions, although MeCP2 was not associated with any clinical parameters. The expression of claudin-6 was not significantly correlated with the expression of MeCP2, just as Wojdacz' s research, the methylation of breast cancer related genes (BRCA1, APC and RASSF1A) in peripheral blood DNA did not directly link to somatic methylation of the same genes in tumor DNA [31]. But there also were 74 cases of breast IDC in which the expression of claudin-6 and MeCP2 showed a reverse trend, suggesting that the increased expression of MeCP2 also played an important role in the progression of breast IDC. Similarly, MeCP2 mRNA expression levels have be shown to be increased in breast cancer specimens [32]. In addition to binding methylated DNA sequences, MeCP2 contains a C-terminal transcriptional repression domain which has been identified as the region involved in the gene repression activity [33]. MeCP2 binds methylation of the breast cancer 1 gene (*BRCA1*) and MAGE-A promoter, and results in tumor suppression [34,35]. Our group has previously found that MeCP2 can bind the promoter of claudin-6 in breast cancer cell line MCF-7 (data unpublished), A number of studies have shown that MeCP2 is responsible for the initial recruitment of HDAC1, Sin3A and HDAC2, forming a tumor suppressor complex and were essential for MeCP2-mediated tumor suppression [36,37].

Histone deacetylases are modification enzymes that catalyze the removal of acetyl molecules from lysines to balance the activities of histone acetyl-transferases [38]. Previous studies have shown that HDAC1 was overexpressed in many cancers, including gastric [39], colorectal [40] and pancreatic [41] carcinomas. To investigate the interactions between claudin-6 and HDAC1, H3Ac and H4Ac, we evaluated the expression patterns and the relation among the HDAC1, H3Ac and H4Ac in the breast IDC. In our series, HDAC1 expression was increased in IDC specimens relative to breast FA specimens. Interestingly, our data suggested that up-regulation of HDAC1 favored tumor cell metastasis, as evidenced by the fact that those tumors showing increased HDAC1 expression correlated with poor differentiation, older age, lymph node metastasis. Therefore, our data indicate that higher expression levels of HDAC1 are correlated with more aggressive tumors. This finding was not consistent with the observations of Krusche et al [42], who showed that HDAC1 expression predicted a better prognosis. The reasons for these results is unknown and the clinical significance of HDAC1 expression at the protein level in breast IDC also needs to be confirmed in a larger patient cohort. The Spearmen's relation test showed the expression of HDAC1 was not correlated with the expression of claudin-6, but there also were 64 cases of breast IDC in which the expression of claudin-6 and HDAC1 showed a reverse trend, suggesting that there was a partial role of increased expression HDAC1 in repressing the expression of claudin-6. Histone modifications (e.g. acetylation, methylation) are important regulators of transcriptional activities; therefore, we evaluated the total histone H3 acetylation (H3Ac) and histone H4 acetylation (H4Ac) by immunohistochemistry. In the present study, H3Ac expression levels were markedly increased in IDC specimens compared to breast FA specimens. H3Ac levels were increased in tumors < 5 cm in size and cancers of clinical stage I and II. H4Ac protein expression was high in IDC. However, we found no statistically significant correlations between H4Ac and clinicopathological parameters. We did not find statistically significant correlation between claudin-6 and H3Ac, H4Ac. These results therefore suggest that DNA methylation and histone modification play only a partial role in inhibition of claudin-6 expression. MeCP2, DNMT1, HDAC1, H3Ac and H4Ac might form a repressor complex, and inhibit the expression of claudin-6. Whether or not such complex might be responsible for the down-regulation of claudin-6 observed in our cases would require further investigation. Interestingly, Spearman's correlation test showed the expression of MeCP2 was positively correlated with the expression of H3Ac and H3Ac expression positively correlated with the expression of H4Ac. These results indicate that the expression of DNA methylation associated gene (MeCP2) may influence the expression of histone acetylation (H3Ac and H4Ac) in breast IDC and that the expressions of MeCP2, H3Ac and H4Ac play an important role in the generation of breast IDC.

We would have liked to include a survival analysis of the IDCs, but the breast cancer cases were mostly from the period 2009-2010, only two or three years after surgery. The 5-year survival rate seems to be more meaningful for assessment than nodal metastasis. This research is being conducted now and we hope to be able to publish the data in the future.

## Conclusions

In summary, our data indicates that claudin-6 is down-regulation in breast IDC and that it is mediated by molecular mechanisms other than aberrant expression of the methylation associated proteins (MeCP2, DNMT1) and histone modification associated proteins (HDAC1, H3Ac, H4Ac). The expression of MeCP2 is positively correlated with the expression of H3Ac and H4Ac. Our data suggests that down-regulation of claudin-6 is an important factor influencing lymphatic metastasis; whereas up-regulation of HDAC1 is associated with tumor progression and invasiveness in breast IDC.

## Abbreviations

MeCP2: Methy-CpG binding protein 2; DNMT1: DNA methyltransferase 1; HDAC1: Histone deacetylase 1; H3Ac: Acetyl-histone H3; H4Ac: Acetyl-histone H4; IDC: Invasive ductal carcinoma; FA: Fibroadenoma.

## Competing interests

The authors declare that they have no competing interests.

## Authors' contributions

CQ and XX carried out most of experiments, participated in the design of the study, performed the statistical analysis, and drafted the manuscript. HJ, YL and LL participated in the design of the study and helped draft the manuscript. QW, YG, LY, and ZL assisted the experiments. TZ, XZ, and XD participated in the study design and coordination. All authors have read and approved the final manuscript.
